# Cereblon-Recruiting PROTACs: Will New Drugs Have to Face Old Challenges?

**DOI:** 10.3390/pharmaceutics15030812

**Published:** 2023-03-02

**Authors:** Marcin Cieślak, Marta Słowianek

**Affiliations:** 1 Department of Food Science, Faculty of Pharmacy, Medical University of Lodz, ul. Muszynskiego 1, 90-151 Lodz, Poland; 2 Centre of Molecular and Macromolecular Studies, Polish Academy of Sciences, 112 Sienkiewicza Str., 90-363 Lodz, Poland

**Keywords:** PROTAC, protein degrader, IMiD, thalidomide, cereblon, E3 ubiquitin ligase, cancer, resistance

## Abstract

The classical low-molecular-weight drugs are designed to bind with high affinity to the biological targets endowed with receptor or enzymatic activity, and inhibit their function. However, there are many non-receptor or non-enzymatic disease proteins that seem undruggable using the traditional drug approach. This limitation has been overcome by PROTACs, bifunctional molecules that are able to bind the protein of interest and the E3 ubiquitin ligase complex. This interaction results in the ubiquitination of POI and subsequent proteolysis in the cellular proteasome. Out of hundreds of proteins serving as substrate receptors in E3 ubiquitin ligase complexes, current PROTACs recruit only a few of them, including CRBN, cIAP1, VHL or MDM-2. This review will focus on PROTACs recruiting CRBN E3 ubiquitin ligase and targeting various proteins involved in tumorigenesis, such as transcription factors, kinases, cytokines, enzymes, anti-apoptotic proteins and cellular receptors. The structure of several PROTACs, their chemical and pharmacokinetic properties, target affinity and biological activity in vitro and in vivo, will be discussed. We will also highlight cellular mechanisms that may affect the efficacy of PROTACs and pose a challenge for the future development of PROTACs.

## 1. Introduction 

Tumorigenesis is regulated by a number of proteins that are aberrantly expressed, many of which facilitate tumor progression (proto-oncogenes), while others act as tumor suppressors. In healthy tissues, numerous proto-oncogenic proteins are degraded in the ubiquitin-proteasome system (UPS), thus avoiding tumor development. The UPS regulates a wide variety of cellular processes through ubiquitination of specific substrate proteins and subsequent degradation by the 26S proteasome. The ubiquitination process requires the orchestrated action of three enzymatic complexes: an E1-enzyme that activates ubiquitin, an E2 ubiquitin-conjugating enzyme and an E3 ubiquitin ligase. The selectivity of this process depends on the recognition of the target protein by the E3 ubiquitin ligase [1]. 

In recent years, UPS has been used as the therapeutic strategy to remove pathogenic proteins at the post-translational level. For this purpose, synthetic, low-molecular-weight compounds called proteolysis targeting chimeras (PROTACs) have been developed. These heterobifunctional molecules contain two different ligands connected by a linker. One ligand recruits E3 ubiquitin ligase while the other ligand binds the protein targeted for degradation (protein of interest—POI). PROTACs induce the physical proximity between the POI and the E3 ubiquitin ligase, and thus facilitate ubiquitination of the target protein and its subsequent degradation by 26S proteasome [2]. The idea of PROTACs was pioneered by Deshaies and Crews in 2001 [3]. Currently designed PROTACs recruit only a few of many E3 ligases, mainly cereblon (CRBN), mouse double minute 2 (MDM2), inhibitor of apoptosis proteins (cIAPs), and Von Hippel–Lindau (VHL) [4,5]. 

**Figure 1:**
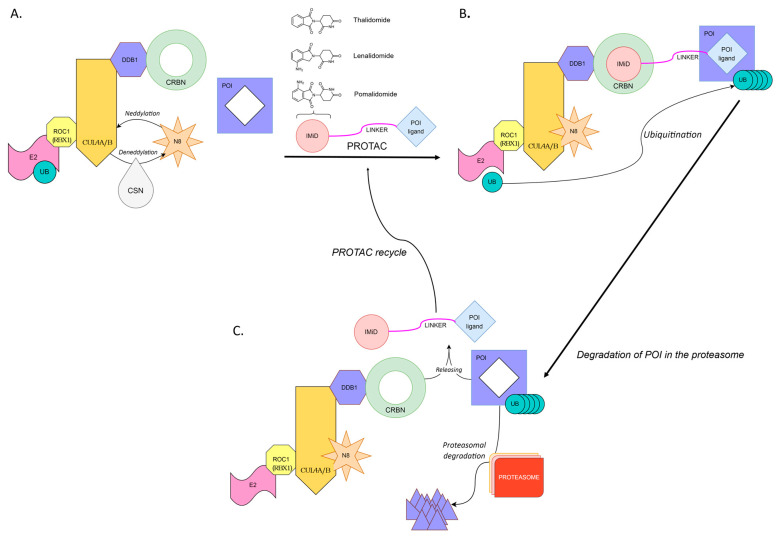
PROTAC-induced degradation of a target protein. (**A**) Neddylation by N8 (NEDD8) activates Cullin-RING E3 ligase. PROTAC consists of a ligand for the target protein (POI) connected by a linker with an E3 ligase binder (IMiD). (**B**) PROTAC-induced connection between POI and E3 ligase leads to ubiquitin transfer from E2 to POI. (**C**) The polyubiquitinated POI is recognized and degraded in the proteasome. The PROTAC is finally released and can catalyze the next round of ubiquitination of POI. CRBN, Cereblon; CSN, COP9 signalosome, responsible for deneddylation of Cullin-RING E3 ligases; N8 (NEDD8), Neural-precursor-cell-expressed developmentally down-regulated protein 8; CUL4A/B, Cullin 4A or Cullin 4B protein from cullin family of ubiquitin ligase proteins; DDB1, Damage-specific DNA binding protein 1; E2, Ubiquitin-conjugating enzyme; E3,Ubiquitin ligase; IMiD, Immunomodulatory drug e.g., thalidomide, lenalidomide, pomalidomide; POI: protein of interest, the protein that is marked for degradation; POI ligand, ligand for POI; PROTAC: Proteolysis targeting chimera; PROTEASOME, Protein complexes degrading cellular proteins by proteolysis; ROC1 (Rbx1/Hrt1), RING family protein, E2 binding subunit of E3 ligase; UB, Ubiquitin.

CRBN E3 ubiquitin ligase (CUL4-RBX1-DDB1-CRBN or Cullin4-RING ligase (CRL4^CRBN^)) is composed of CRBN, damaged DNA-binding protein 1 (DDB1), Cullin4 (Cul4A/4B) and regulator of cullins 1 (RBX1). CRBN is responsible for the recognition and binding of the substrate protein(s) targeted for ubiquitination, while DDB1 serves as a platform protein linking Cul4 to CRBN [5,6]. The discovery that immunomodulatory imide drugs (IMiDs) bind CRBN, enabled the development of CRBN-recruiting PROTACs (or IMiD-PROTACs) [7,8,9]. Since then, many CRBN-recruiting PROTACs have been designed to target nuclear receptors, protein kinases, transcriptional factors, regulatory proteins, neurodegeneration-related proteins or cellular metabolic enzymes [10]. PROTAC-induced protein degradation is schematically depicted in Figure 1. 

Many in vitro and in vivo studies have shown that PROTACs are potent tools in regulating protein expression. The clinical evaluation of PROTACs began in 2019. Currently, 15 phase I/II clinical studies are being conducted to test the efficacy of PROTACs in cancer (fourteen trials) and autoimmune diseases (one trial). Interestingly, out of fifteen clinically tested PROTACs, twelve recruit CRBN E3 ligase, one recruits VHL E3 ligase and two PROTAC structures are unknown (Table 1) [11,12]. However, as with all anticancer drugs, the chronic use of IMiD-PROTACs will eventually result in the selection of resistant cancer cells. 

This article presents CRBN-recruiting PROTACs developed in the last few years and summarizes their biological activity. In Section 2, we review CRBN-recruiting PROTACs targeting BET proteins, cyclin-dependent kinases (CDK6 and CDK9), Bruton’s tyrosine kinase, Akt, STAT transcription factors, MCL-1, BCL-1, Anaplastic lymphoma kinase, Androgen receptor, TrkC, BCR-ABL, Sirtuin 2, Histone deacetylase 6, EGFR, GSPT1/2, TGFβ1 and CYP1B1. The biological activity and potency, as well as chemical structures of some PROTACs are presented. In Section 3, we discuss factors that may affect the clinical efficacy of PROTACs, in particular the expression of CRBN, DDB1 and CUL4, and mutations of CRBN. Possible mechanisms of resistance to PROTACs are reviewed in Section 4.

## 2. Overview of Cereblon-Recruiting PROTACs

### 2.1. Bromodomain and Extra-Terminal Domain Proteins (BET)

The BET family includes four proteins of about 110 amino acids located in the cell nucleus. BRD2, BRD3 and BRD4 are widely expressed across different cell types, while BRDT is expressed only in the male germ cells [13,14]. BRD4 affects cancer-cell proliferation by regulating the expression of oncogenes such as c-myc, Bcl-xL, Bcl-6 or cyclin D1(2) and is overexpressed in many types of human cancers. Although traditional small-molecule BRD4 inhibitors have shown promise in the treatment of Myc-driven cancers such as Burkitt’s lymphoma (BL), their use often leads to accumulation of the BRD4 protein, which results in limited suppression of Myc expression, low antiproliferative activity and no induction of apoptosis. These limitations have been overcome by BET PROTACs.

The first PROTAC targeting the BRD2/3/4 proteins was described by Winter et al. dBET1 is composed of the bromodomain inhibitor JQ1 conjugated with thalidomide to recruit CRBN E3 ligase. dBET1 induced the degradation of the BRD4 in vitro and in vivo, which in turn led to the downregulation of PIM1 and MYC proteins. Studies in AML cells and in a mouse xenograft model of AML showed that anticancer activity of dBET1 was higher than JQ1. A 14-day treatment of tumor-bearing mice (human MV4;11 leukemia xenograft) with dBET1 resulted in a significant reduction in tumor size [15].

Another example of BRD4-targeting PROTAC is ARV-825, which consists of the BRD4 inhibitor OTX015 conjugated to pomalidomide. ARV-825 efficiently catalyzed the degradation of BRD4, which led to a downregulation of c-MYC, PIM1, CDK 4/6, JAK2 and Bcl-xL. However, this PROTAC also induced complete degradation of the neosubstrates IKZF1 and IKZF3. Studies in BL cell lines showed superiority of ARV-825 over BRD4 inhibitors (JQ1 and OTX015) in the inhibition of the proliferation and activation of apoptosis. A 50% degradation of BRD4 was observed after a 2 h treatment of BL cells with ARV-825 at a concentration of 100 nM. ARV-825 at 10 nM led to almost complete degradation of BRD4 within 6 h, with maintenance of the BRD4 degradation effect up to 24 h. DC50 values < 1 nM were achieved after overnight incubation of cells with ARV-825 [16,17].

Similar effects were observed in thyroid cancer, where ARV-825 inhibited cell survival, proliferation and migration of the primary human thyroid cancer cells and TPC-1 cell line. ARV-825 induced BRD4 protein degradation and the down-regulation of its targets (c-Myc, Bcl-xL and cyclin D1). In vivo studies showed potent inhibition of TPC-1 tumor growth in immunodeficient mice after oral administration of ARV-825 [18].

ARV-825 efficiently inhibited proliferation and induced apoptosis of T-ALL cells by degrading the BET proteins and suppressing downstream targets such as c-Myc, in vitro and in vivo. DC50 for BRD2, BRD3 and BRD4 was in the nanomolar range. As an inhibitor of proliferation, ARV-825 showed superiority over BET protein inhibitors (JQ1, OTX015) and dBet1 PROTAC. In a xenograft model of T-ALL (CCRF cells), ARV-825 significantly reduced tumor growth [19].

Zhang et al. proposed new BRD4 protein degraders consisting of a dihydroquinazolinone-based BRD4-inhibitor and lenalidomide/pomalidomide for the recruitment of CRBN E3 ligase. They identified PROTAC 21, which was four-fold more potent in inhibiting THP-1 cell line growth (IC50 0.81 µM) than BRD4 inhibitor (IC50 3.26 µM). PROTAC 21 showed excellent activity in BRD4 BD1 inhibition (IC50 41.8 nM) and induced BRD4 degradation and c-Myc suppression at a concentration of 1 µM [20].

Zhou et al. synthesized ZBC260 PROTAC composed of the high affinity BET inhibitor HJB97 and lenalidomide moiety. ZBC260 induced degradation of BRD2, BRD3 and BRD4 at concentrations as low as 0.1–0.3 nM and downregulated c-Myc at 0.1 nM in the acute leukemia cell line RS4;11. Downregulation of c-Myc protein by BET degrader was 1000-fold more potent compared to the BET inhibitor HJB97. ZBC260 inhibited the growth of acute leukemia cells RS4;11 and MOLM-13, showing IC50 values of 51 pM and 2.3 nM, respectively. A pharmacodynamic study in mice bearing RS4;11 xenograft tumors showed more than 90% tumor regression after ZBC260 administration with a well-tolerated dosing regimen (at 5 mg/kg intravenously, three times a week for 3 weeks). A pharmacokinetic analysis showed that maximum concentrations of the degrader in plasma and tumor tissue were 392 ng/mL and 166.3 ng/g, respectively, 1 h after drug administration [21]. The high efficacy of ZBC260 against triple-negative human breast cancer in vitro and in vivo was demonstrated by Bai et al. [22].

In 2018, Qin et al. designed a QCA570 PROTAC composed of a novel class of BET inhibitor 1,4 oxazepine (QCA276) and lenalidomide. QCA570 was a more potent inhibitor of leukemia cell growth, compared to other BET PROTACs (dBET1, ARV-825, ARV-771, ZBC260). Complete and long-term tumor regression was demonstrated in both MV4;11 and RS4;11 xenograft models of acute leukemia in mice at a dose of 5 mg/kg or 1, 2.5, 5 mg/kg administered 3 times per week, for 2 or 3 weeks, respectively. Pharmacokinetic analysis in mice bearing RS4;11 xenograft tumors revealed that 1h after a single intravenous dose of 5 mg/kg, the highest plasma concentration of QCA570 was 2360 ng/mL, which gradually decreased to be undetectable after 6h. In conclusion, QCA570 can be considered the most potent and effective BET protein degrader described to date [14].

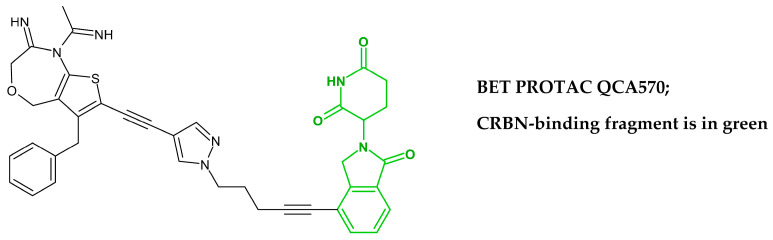



BRD2, BRD3 and BRD4 play important roles in transcriptional regulation. However, selective drugs for each of these proteins are unavailable, which may lead to undesirable side effects following therapy. Jiang et al. synthesized BET degraders consisting of a novel inhibitor benzo[cd]indole-2-one selective towards the first BET bromodomain (BD1), and thalidomide as a ligand for CRBN. The inhibitor benzo[cd]indole-2-one exhibited 50-fold higher inhibitory activity against BD1 BRD4 (IC50 49.5 nM) than against BD2 BRD4 (IC50 2479 nM). Degrader 15 catalyzed the selective removal of BRD4 and BRD2, and effectively inhibited cell growth of human acute leukemia, breast cancer and non-small-cell lung cancer, with the GI50 values in the nanomolar range. PROTAC 15 induced the complete degradation of BRD4 and c-Myc proteins in MV4-11 cells at a concentration of 50 nM. This is the first example of PROTAC selectively targeting BRD4 and BRD2 proteins [13].

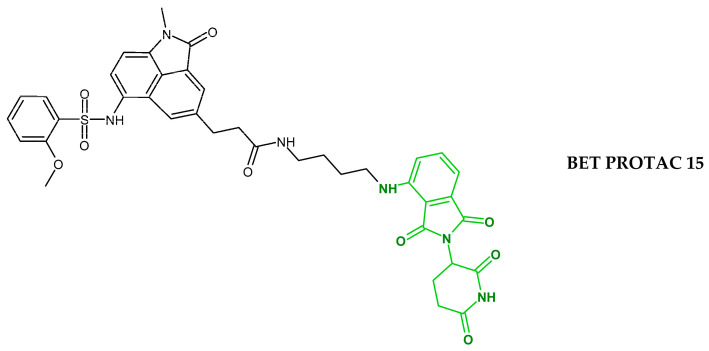



### 2.2. Cyclin-Dependent Kinases

Cyclin-dependent kinases CDK4 and CDK6,together with cyclin D, play a key role in regulating the cell’s transition between G1 and S phase during mitosis. CDK4 and CDK6 kinase inhibitors such as palbociclib, ribociclib and abemaciclib have become important FDA-approved drugs for breast-cancer patients [23]. Anderson et al. (2020) developed and tested palbociclib-based PROTACs containing ligands for different E3 ligases (VHL, CRBN, cIAP) that effectively degraded CDK4 and CDK6 in the Jurkat acute lymphoblastic leukemia cells. CRBN—recruiting PROTAC 10^15^ was the most potent CDK6 degrader, with a pDC50 value of 9.1. This PROTAC also induced the degradation of CDK4, with a pDC50 value of 8.0 [24].

Su et al. designed a series of CDK6 degraders by coupling CDK4/6 inhibitors (palbociclib, ribociclib and abemaciclib) and ligands to recruit CRBN-, MDM2-, VHL- and cIAP-E3 ligases (i.e., pomalidomide, nutlin-3b, VH032 and bestatin, respectively). The best activity was demonstrated by CP-10 PROTAC composed of palbociclib and pomalidomide, which induced the degradation of 89% of CDK6 at 100 nM in human glioma U251 cells. The CP-10-induced degradation of CDK4 was 50~80-fold weaker than CDK6 (DC50 values of 180 nM and 2.1 nM, respectively). CP-10 showed significantly higher antiproliferative activity in multiple myeloma MM.lS (IC50 10 nM) and mantle cell lymphoma Mino cells (IC50 8 nM), compared to palbociclib (IC50 200 nM and 45 nM, respectively). In addition, CP-10 potently degraded mutated CDK6 (D163G and S178P), enabling its clinical application in drug resistance [25]. 

CDK9 is involved in the transcriptional elongation of several target genes, is widely expressed, and contributes to various cancers such as pancreatic, prostate and breast cancer. Thus, the degradation of CDK9 or the inhibition of kinase activity may restore the ability of cancer cells to undergo apoptosis. Wogonin is a natural compound isolated from *Scutellaria baicalensis*, and has been shown to be a potent and selective CDK9 inhibitor, similar to flavopiridol (Alvocidib), which was the first CDK9 inhibitor involved in clinical trials. Using a ‘click chemistry’ approach, a series of 8 CRBN-recruiting PROTACs targeting CDK9 were synthesized. The PROTACs contained wogonin as a ligand for CDK9 and pomalidomide to recruit CRBN E3 ligase. The results of cellular assays showed that PROTACs containing a triazole group in the linker selectively downregulated the intracellular level of CDK9. Among them, PROTAC 11c induced the selective degradation of CDK9 in MCF-7 cells, with no effects on CDK2, CDK4, CDK5, CDK7 and CDK8 [26].

Another successful attempt to obtain a PROTAC selective for CDK9 was by Robb et al. They synthesized a degrader 3 composed of the aminopyrazole inhibitor of CDK9 and thalidomide. Degrader 3 selectively downregulated CDK9 in HCT116 cells without affecting other CDK kinases (CDK2 and CDK5), as well as kinases such as IKKβ, Akt and FAK. Quantitative Western blot analysis showed that at a concentration of 20 µM, degrader 3 reduced CDK9 levels by 65%, thus becoming a novel tool that can be used to analyze the role of CDK9 in various disease states [27].

Potential PROTACs targeting CDK9 were obtained by coupling the selective CDK9 inhibitor BAY-1143572 via different linkers (PEG or alkyl triazoles) to pomalidomide. PROTAC B03 displayed high degradation efficiency, with a DC50 value of 7.62 nM, and showed 20-fold more potent antiproliferative activity (IC50 25 nM) than the CDK9 inhibitor BAY-1143572 (IC50 0.56 µM) in MV4-11 leukemia cells. Degrader B03 was highly selective against five of 403 kinases, including CDK9, as evaluated by the DiscoverX KINOME scan. In MV4-11 cells, B03 selectively degraded CDK9 without any effect on CDK1, CDK2, CDK6 and CDK7. PROTAC B03 induced CDK9 degradation in vivo in mouse models of acute myeloid leukemia (AML,) and showed acceptable pharmacokinetic properties with a plasma half-life of more than 1.3h after a single intravenous injection of 5 mg/kg [28].

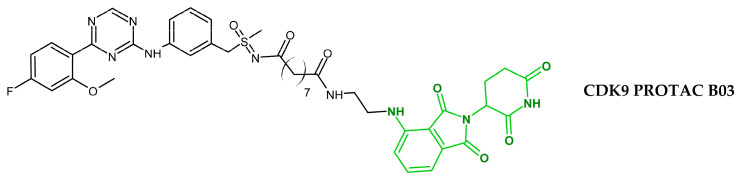



### 2.3. Bruton’s Tyrosine Kinase (BTK)

BTK plays a key role in B-cell development, regulating their growth, maturation, migration and apoptosis. Dysregulation of any of these pathways is associated with conditions such as cancer, autoimmunity and inflammation. BTK activates multiple pro-survival and proliferative pathways, including the activation of PLCγ-2 to release intracellular calcium stores, as well as the Ras/Raf/MEK/ERK kinase pathway [29]. Patients with chronic lymphocytic leukemia (CLL) or other B-cell cancers are treated with ibrutinib—an irreversible inhibitor of BTK. However, due to a cysteine-to-serine substitution (C481S) in the ibrutinib binding site of BTK, more than 80% of CLL patients acquire resistance [30]. 

PROTAC MT-802 induces BTK degradation of both the wild-type and the C481S mutant. It is composed of ibrutinib derivative connected to pomalidomide by a polyethylene glycol (PEG) linker. MT-802 showed better selectivity compared to ibrutinib, and exhibited potent degradation activity against wild-type and mutant BTK (C481S) (Dmax > 99%, DC50 9.1 nM in NAMALWA cells). MT-802 also reduced the amount of phosphorylated BTK in cells isolated from CLL patients with the C481S mutation [31].

The replacement of pomalidomide in MT-802 by a lenalidomide analogue resulted in a novel PROTAC SJF620, which induced potent BTK degradation in cells (DC50 7.9 nM, Dmax 95%). Moreover, SJF620 had an improved pharmacokinetic profile compared to MT-802 in mouse studies, showing robust clearance (Cl 40.8 mL/min/kg) and exposure (AUClast 405 min*ng/mL) with a half-life (t ½ 1.62 h) [32].

Xue et al. (2020) synthesized a series of degraders targeting BTK composed of the ibrutinib or PLS-123 (BTK inhibitors) and pomalidomide or VH-032 (ligands for CRBN- and VHL E3 ligase, respectively). PROTAC 6b composed of pomalidomide and PLS-123 effectively degraded BTK protein with a DC50 less than 300 nM and a Dmax of 75%, at a concentration of 1 µM [33].

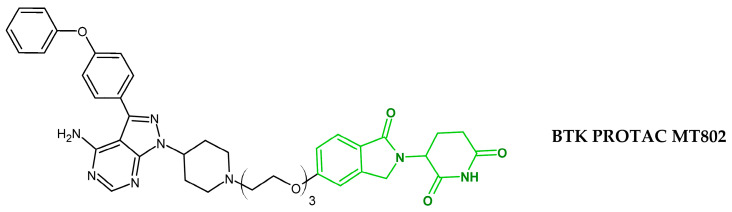



### 2.4. AKT

The Akt family of serine/threonine protein kinases includes three highly homologous isoforms, Akt1, Akt2 and Akt3, encoded by different genes. Increased expression and activation of this kinase has been observed in many cancers, including ovarian, lung, pancreatic, glioma, head and neck, gastric, prostate and breast cancer. Akt contributes to cancer progression by inhibiting apoptosis and promoting angiogenesis, changes in metabolism, cell proliferation, migration and invasion [34].

INY-03-041 is a pan-AKT degrader downregulating all three AKT isoforms. Its structure contains GDC-0068 (Akt inhibitor) conjugated via a 10-hydrocarbon linker to a lenalidomide. The inhibitory activity of INY-03-041 for Akt1 (IC50 2.0 nM), Akt2 (IC50 6.8 nM) and Akt3 (IC50 3.5 nM) was comparable to GDC-0068 (IC50 values of 5, 18 and 8 nM, respectively). However, Akt PROTAC showed much better inhibition of the growth of cancer cells compared to the Akt inhibitor alone. The most potent effect was observed in ZR-75-1 cells (GI50 16 nM), with 14-fold increased potency compared to GDC-0068 (GI50 229 nM). At 100 and 250 nM, INY-03-041 induced the degradation of all three AKT isoforms in the breast cancer cell line MDA-MB-468, and reduced the levels of the direct AKT substrates—phosphorylated PRAS40 (pPRAS40) and GSK3β (pGSK3β). A ring-finger protein RNF166 (a known target of lenalidomide), IKZF1 and IKZF3 were identified as neosubstrates of INY-03-041 [35].

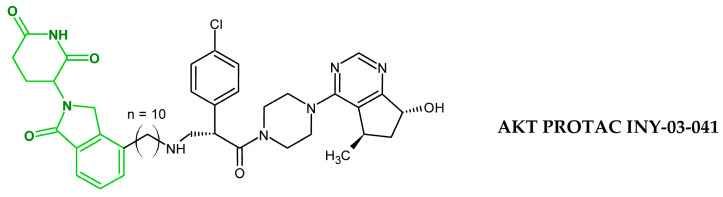



### 2.5. STAT Transcription Factors

STAT3 transduces signals from receptors on the cell surface to the nucleus. Sustained activation of STAT3 promotes the growth, survival and metastasis of cancer cells, and inhibits the anti-tumor immune response [36]. 

SI-109 is a low-molecular-weight compound that binds to the SH2 domain of STAT3 and blocks its function. Using SI-109 and IMiDs (lenalidomide or pomalidomide), a series of PROTACs recruiting CRBN-E3 ligase were synthesized. SD-36 was identified as the most potent and selective STAT3 degrader. SD-36 induced proteolysis of STAT3 and pSTAT3Y705 with DC50 of 28 nM and 60 nM in SU-DHL-1 (lymphoma) and MOLM-16 (leukemia) cells, respectively. SD-36 showed more than 100-fold selectivity in degrading STAT3 over other STAT proteins. This degrader inhibited the growth of MOLM-16 and SU-DHL-1 cells (GI50 of 13 nM and 610 nM, respectively) and induced complete tumor regression in the mice model of MOLM-16 xenograft. A single 50 mg/kg dose of SD-36 reduced the levels of STAT3 and pSTAT3Y705 by > 95% in the mice tumor tissue of MOLM-16 xenografts [37].

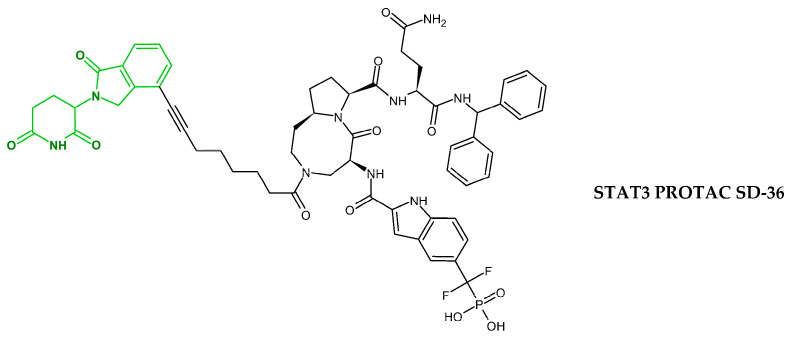



### 2.6. MCL-1 and BCL-2

MCL-1 (myeloid cell leukemia sequence 1) and BCL-2 (B-cell lymphoma 2) prevent apoptosis by binding to proapoptotic proteins, i.e., Bim, Bak and Bax. Overexpression of MCL-1 is frequently observed in many types of malignancies, including solid tumors and hematological malignancies such as acute myeloid leukemia (AML) and multiple myeloma (MM). Mcl-1 has been linked to tumorigenesis, poor clinical prognosis and drug resistance, and its levels directly correlate with disease progression [38]. 

Mcl-1 and Bcl-2 inhibitors (S1-6 and Nap-1, respectively) were used for the synthesis of Mcl-1 and Bcl-2 PROTACs (C3 and C5, respectively) recruiting CRBN E3 ligase. C3 and C5 induced the degradation of Mcl-1 and Bcl-2 in Hela cells with DC50 values of 0.7 μM and 3.0 μM, respectively. Furthermore, the degrader C3 showed significantly higher cytotoxicity (CC50 0.03 µM) against the Mcl-1-driven H23 cell line, compared to the Mcl-1 inhibitor (A-1210477). C3 and C5 showed reversible depletion of Bcl-2 and Mcl-1 in cells, and can be considered as new tools to investigate the function of these proteins in apoptosis [39].

Papatzimas et al. (2019) developed a novel dMCL1-2 PROTAC that effectively degraded Mcl-1 at nanomolar concentrations. dMCL1-2 is composed of the Mcl-1 inhibitor (A-1210477), a piperazine linker and 4-hydroxythalidomide. dMCL1-2 showed high affinity for Mcl-1 protein, with Kd of 30 nM. A significant downregulation of Mcl-1 protein level was observed in OPM2 cells treated with dMCL1-2 at 100 nM. At 250 nM, dMCL1-2 induced apoptosis in OPM2WT cells, as demonstrated by caspase-3 cleavage. Studies in CRBN knockout cells or with bortezomib demonstrated that the activity of dMCL1-2 was dependent on CRBN and the proteasome. In preliminary metabolic-stability studies using human liver microsomes, both A-1210477 and dMCL1-2 showed similar t1/2 values, of 21.7 and 20.6 min., respectively [40].

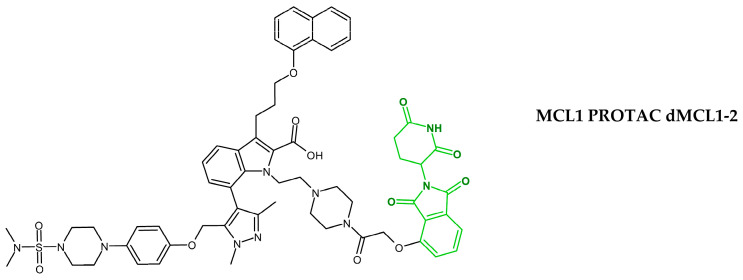



Inhibitors of the anti-apoptotic protein BCL-X_L_ (for example A1155463 or ABT-263 (navitoclax)) have the potential to be used as anticancer drugs. However, they lack selectivity toward cancer cells, and their use is associated with significant toxicity in human blood platelets. Using these inhibitors, Zheng et al. developed a series of PROTACs (XZ424, XZ739) which selectively downregulated BCL-X_L_ in MOLT-4 leukemia cells but not in platelets. PROTAC XZ739 potently downregulated BCL-X_L_ in MOLT-4 cells (DC50 of 2.5 nM after 16 h) and reduced their viability (IC50 10.1 nM), while being much less toxic against platelets (IC50 1.2 µM). This example shows that PROTAC technology can minimize the potential side effects (i.e., thrombocytopenia) resulting from the use of BCL-X_L_ inhibitors only [41,42]. 

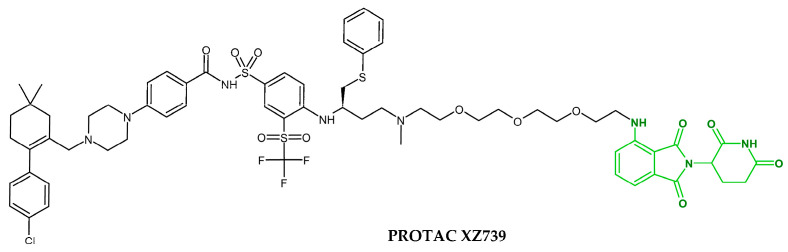



BCL-X_L—_targeting PROTACs can also be used as senolytic (anti-aging) agents by inducing the apoptosis of senescence cells (SCs), which play a role in the development of age-related pathologies. Studies in mice showed that PROTAC PZ15227 (structurally similar to XZ739) efficiently eliminated SCs in bone-marrow stromal cells and hematopoietic stem cells, and reduced the effects of aging in bone and hematopoietic tissues [43]. In another study, Zhang et al. demonstrated that PROTAC SIAIS361034 effectively downregulated BCL-X_L_, leading to the inhibition of the Hedgehog pathway in vitro and in vivo [44].

### 2.7. ALK

Anaplastic lymphoma kinase (ALK) is a receptor tyrosine kinase, belonging to the insulin-receptor kinase subfamily. Aberrant activation ALK has been linked to many types of human cancer, such as anaplastic large-cell non-Hodgkin’s lymphoma (ALCL), non-small-cell lung cancer (NSCLC), neuroblastoma, renal cell carcinoma (RCC), and thyroid and breast cancer. The FDA has so far approved four ALK inhibitors (crizotinib, ceritinib, alectinib, brigatinib) for the treatment of patients with ALK-positive NSCLC [45]. To overcome the problem of drug resistance occurring in patients treated with these inhibitors, research is being conducted into compounds with novel mechanisms of action. 

In 2018, Zhang et al. synthesized two PROTACs, MS4077 and MS4078, by coupling ceritinib to pomalidomide through two different linkers. MS4077 and MS4078 potently reduced the levels of oncogenic active ALK fusion proteins and inhibited ALK and STAT3 phosphorylation in SU-DHL-1 lymphoma and NCI-H2228 lung cancer cells. MS4077 and MS4078 induced the degradation of ALK fusion proteins in SU-DHL-1 cells (DC50 values of 3 nM and 11 nM, respectively), and in NCI-H2228 cells (DC50 values of 34 nM and 59 nM, respectively). MS4077 and MS4078 showed high affinity for ALK (Kd 37 nM and 19 nM, respectively). Furthermore, these compounds inhibited SU-DHL-1 cell proliferation (IC50 46 nM for MS4077 and 33 nM for MS4078). The activity of both PROTACs were dependent on CRBN and proteasome. In mouse pharmacokinetic studies, compound MS4078 showed good plasma exposure. After 2h of drug administration, the highest plasma concentration was 3000 nM, which decreased to 340 nM after 12h. At a dose of 50 mg/kg, MS4078 was well tolerated, and no adverse reactions were observed [46].

Yan et al. investigated the anticancer activity of ALK PROTAC in vivo. They tested a series of degraders composed of the ALK inhibitor LDK378 (ceritinib) coupled by different linkers to pomalidomide, and identified compound B3 as the most efficient ALK degrader. The treatment of H3122 cells with PROTAC B3 at a concentration of 50 nM resulted in the significant degradation of EML4-ALK and p-ALK fusion proteins and the downregulation of p-STAT3. B3 demonstrated activity against cancer cells with mutated ALK (L1196M and G1202R) conferring resistance to crizotinib. In H3122 xenograft mouse models, B3 at doses of 25 mg/kg and 50 mg/kg inhibited tumor growth by 37% and 48%, respectively. No significant weight loss or toxicity was observed, suggesting the safety of B3 [47].

Two ALK degraders, TL13-12 and TL13-112, were developed by coupling pyrimidine-based ALK inhibitors, TAE684 or ceritinib (LDK378) to the pomalidomide. These PROTACs were highly effective in degrading wild type ALK (DC50 of 10nM in H3122 cells and DC5 40 nM in Karpas 299 cells) and inhibiting the proliferation of anaplastic large cell lymphoma and non-small-cell lung cancer. No degradation of the mutant EML4-ALK fusion protein with resistant mutations L1196M, C1156Y, and G1202R was observed. Proteomic studies showed that these compounds also promoted the degradation of additional kinases (neosubstrates) such as PTK2 (FAK), Aurora A, FER and RPS6KA1 (RSK1) [48].

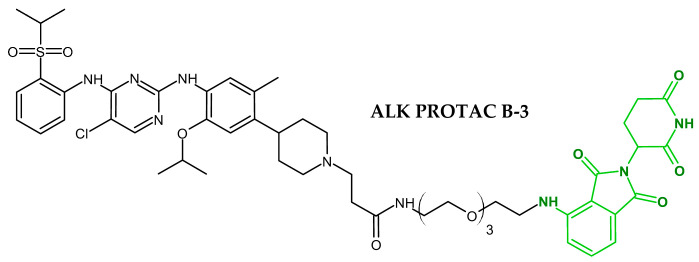



### 2.8. Androgen Receptor 

Androgen receptor (AR) belongs to the superfamily of nuclear hormone receptors. After binding to its natural ligand—the androgen hormone (testosterone, dihydrotestosterone), AR regulates the transcription of target genes. It plays an important role in the development of prostate cancer, and therefore is the main target of therapy [49].

Takwale et al. (2020) evaluated the biological activity of a series of PROTACs targeting the androgen receptor in metastatic prostate cancer cells. The tetramethylcyclobutane-based AR antagonist and thalidomide analogue TD-106 were used for the synthesis of PROTACs. Degrader TD-802 exhibited the highest activity in androgen-dependent LNCaP cells, with a DC50 of 12.5 nM and a Dmax of 93%. TD-802 showed good microsomal stability in mouse liver microsomes, with 96% remaining after 30 min of incubation. In vivo studies in a mouse VCaP xenograft model showed that TD-802 effectively inhibited tumor growth and exhibited a long half-life of >4 h and high plasma exposure after intravenous and intraperitoneal injections [50,51]. 

Han et al. (2021) developed potent degraders by coupling the AR antagonist ARI-16 (aryloxy tetramethylcyclobutane derivative) to thalidomide (ligand for CRBN E3 ligase). PROTAC ARD-2128 efficiently degraded AR in VCaP (DC50 0.28 nM; DC90 3.5 nM) and in LNCaP cells (DC50 8.3 nM; DC90 25 nM), and inhibited the growth of these cell lines with GI50 of 4 nM and 5 nM, respectively. Furthermore, compound ARD-2128 at 10 nM suppressed the expression of AR-regulated genes such as PSA and TMPRSS2 in prostate cancer cells. Pharmacokinetic studies in mice after a single oral administration of ARD-2128 at 20 mg/kg showed high exposure in plasma (1659 ng/mL, after 24 h) and VCAP tumor tissues (1506.7 ng/kg after 24 h), low clearance (1.2 mL/min/kg), long half-life (T1/2 = 18.8 h) and 67% oral bioavailability. Studies in a mouse model of VCaP tumors showed the superiority of degrader ARD-2128 over enzalutamide. Oral administration of ARD-2128 at doses of 10, 20 and 40 mg/kg inhibited tumor growth by 46, 69 and 63%, respectively. No signs of toxicity were observed during the 21-day treatment [52]. 

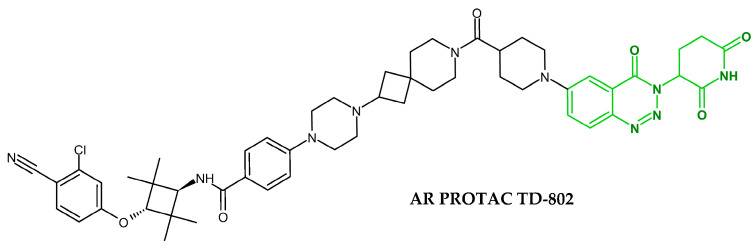



Another example of an AR-targeting PROTAC is ARV-110, which is composed of enzalutamide (a non-steroid AR antagonist which is used in the treatment of castration-resistant prostate cancer) linked to a thalidomide derivative. This PROTAC induced the almost complete (>95%) degradation of AR in VCaP or LNCaP cells (DC50 of around 1 nM). Further studies revealed that ARV-110 inhibited the synthesis of PSA and proliferation of VCaP cells, and also induced their apoptosis. In mouse xenograft models, ARV-110 demonstrated tumor growth inhibition in castrated (almost complete inhibition) and intact (70% inhibition) VCaP models, and in the AR-expressing prostate-patient-derived xenograft (PDX) model. The anticancer activity of ARV-110 in vitro and in vivo was higher than enzalutamide [53,54]. ARV-110 is currently undergoing phase 1 and 2 clinical trials in patients with prostate cancer (Table 1).

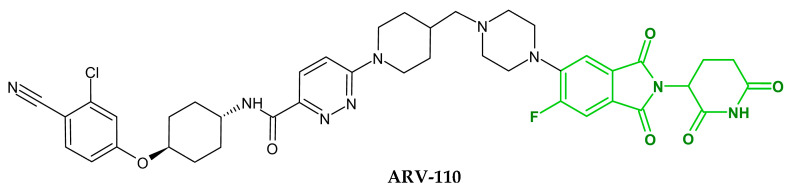



### 2.9. TrkC

Tropomyosin receptor kinase (TrkC) is overexpressed in many types of metastatic cancer, including neuroblastoma, glioma, breast cancer, and melanoma. The activation of TrkC promotes cell growth and metastasis during cancerogenesis. The first TrkC PROTACs were developed by coupling a small-molecule motif IY-IY (TrkC ligand) with pomalidomide (CRBN E3 ligase) or nutlin-3a (MDM E3 ligase). CRBN-recruiting PROTAC induced the degradation of TrkC in the Hs578t cancer cell line, with an estimated DC50 in the range of 0.1–1.0 µM. Interestingly, MDM-recruiting PROTAC exhibited no degradation of TrkC [55].

### 2.10. BCR-ABL

BCR-ABL is an oncogenic fusion protein with excessive tyrosine-kinase activity, involved in the pathogenesis of chronic myeloid leukemia (CML). It drives the process of leukemic transformation and proliferation of neoplastic hematopoietic cells. The BCR/ABL tyrosine kinase phosphorylates more than 20 proteins, resulting in the impaired adhesion of leukemic cells to marrow cells, the infiltration of extra-marrow organs, the inhibition of apoptosis and the loss of cell-cycle control [56].

Lai et al. showed that BCR-ABL can be differentially susceptible to PROTACs-mediated degradation, depending on the type of recruited E3 ligase. BCR-ABL tyrosine kinase inhibitors including imatinib, bosutinib and dasatinib, were used for PROTAC synthesis. Their study demonstrated better efficacy of CRBN-recruiting PROTACs against BCR-ABL when compared to VHL-recruiting PROTACs. For example, bosutinib-CRBN-PROTAC at 2.5 μM induced the degradation of the BCR-ABL protein (>80%), while no effect was observed for bosutinib-VHL-PROTAC. The best degradation of BCR-ABL was observed for DAS 6-2-2-6-CRBN-PROTAC. In the cell survival assay, DAS 6-2-2-6-CRBN showed selective activity against the BCR-ABL-driven K562 line (EC50 of 4.4 nM), while being over 1000-fold less active against non-BCR-ABL-driven cells: HEK293T or SK-BR-3 [57].

Liu et al. (2021) designed and synthesized a series of CRBN-recruiting PROTACs containing dasatinib (a BCR-ABL inhibitor) and pomalidomide or lenalidomide. The PROTACs were subjected to structure–activity-relationship (SAR) studies involving the optimization of linker parameters such as length, hydrophilicity and rigidity. Pomalidomide-based PROTAC 17, containing a linker formed by a sulphur-substituted carbon chain, showed high BCR-ABL degradation activity in vitro (DC50 0.18 nM) and favorable pharmacokinetics in vivo. PROTAC 17 also downregulated several clinically relevant mutant variants of BCR-ABL, including G250E, E255V, V299L, F317L, F317V and T315A. Src kinase was identified as a neosubstrate of this degrader. Ten-day treatment with degrader 17 induced significant tumor regression in a mouse K562 xenograft model. At doses of 1, 3 and 10 mg/kg, tumor growth in mice was inhibited by 78.9%, 93.8% and 98.8%, respectively. Complete tumor regression was achieved at the dose of 10 mg/kg. Furthermore, degrader 17 showed better pharmacokinetic profiles compared to other compounds, with a half-life (T1/2) of 4.3 h [58].

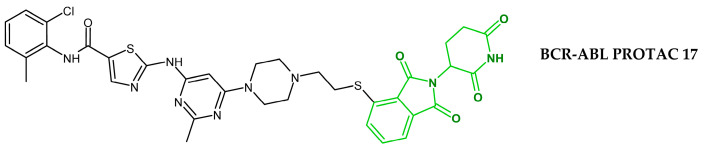



### 2.11. SIRT2 

Sirtuins are proteins belonging to class III lysine deacetylases (KDACs), which remove acetyl groups from lysine residues of many proteins. Thus, sirtuins influence many cellular processes such as transcription, metabolic sensing, inflammation, ageing and apoptosis. The dysregulation of Sirt2 has been linked to the pathogenesis of many diseases, including cancer. The cytoplasmic Sirt2 plays an important role in the cell cycle regulation, autophagy, peripheral myelination, immune and inflammatory responses [59].

Schiedel et al. (2017) synthesized a PROTAC 12 targeting Sirt2, composed of SirReal (a sirtuin-rearranging ligand) and an azido-thalidomide derivative. Such a PROTAC is able to covalently conjugate with alkynylated proteins inside the cell. PROTAC 12 was able to induce the isotype-selective degradation of Sirt2 (Sirt1 levels were not affected) in HeLa cells, resulting in the hyperacetylation of tubulin [60].

### 2.12. HDAC

HDAC6 belongs to the family of histone deacetylases (HDACs) which regulate the acetylation state of cellular proteins. These enzymes are epigenetic erasers, and alter gene expression through the deacetylation of histones. HDAC6 is mainly expressed in the cytoplasm, where it regulates the turnover of misfolded and polyubiquitinated proteins. Th abnormal expression of HDAC6 is associated with cancer development, disease progression, a higher incidence of metastasis and lower survival rates. The overexpression of HDAC6 has been observed in many types of cancers, such as oral squamous cell carcinoma, acute myeloid leukemia, ovarian cancer and hepatocellular carcinoma [61].

The first small-molecule degraders for HDAC6 were developed by Yang et al. (2018), by coupling a non-selective pan-HDAC inhibitor via various linkers to thalidomide analogues. They identified degrader dHDAC6, which showed high activity and selectivity toward the HDAC6 protein in MCF-7 cells (DC50 34 nM and Dmax 70.5%). The mechanism of action of dHDAC6 was dependent on CRBN and proteasome activity. In addition, this degrader also inhibited class I HDACs in the nucleus, as evidenced by increased levels of acetylated histones [62].

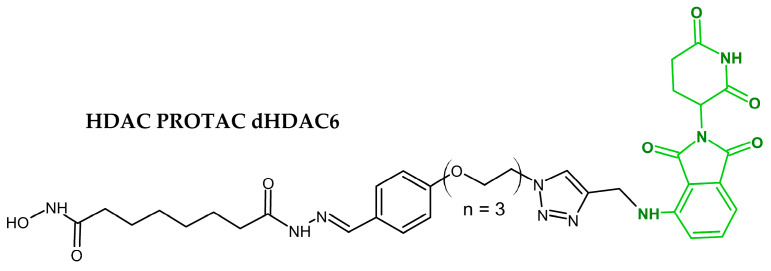



### 2.13. EGFR

The epidermal growth factor receptor (EGFR) is a trans-membrane protein tyrosine kinase that regulates cell proliferation, invasion, metastasis, apoptosis and angiogenesis. Increased EGFR activity was observed in esophagus cancers, glioblastoma, anal cancers, epithelial head and neck cancers, breast cancers and lung cancers [63]. Non-small-cell lung cancer (NSCLC) is often driven by activating mutations in EGFR, i.e., p.L858R or the deletion of exon 19 (Ex19del) [64]. This type of cancer is treated with EGFR tyrosine kinase inhibitors (TKIs); however, the majority of patients acquire resistance due to mutation in EGFR p.T790M [65].

Qu et al. (2021) synthesized two novel CRBN-recruiting PROTACs: SIAIS125 and SIAIS126, consisting of canertinib (an EGFR inhibitor) and pomalidomide. These PROTACs selectively degraded mutant EGFR (L858R+T790M) in H1975 cells (DC50 in the range of 30–50 nM) and EGFR Ex19del in PC9 cells (DC30 30 nM). However, no degradation of wild-type EGFR in A549 lung-cancer cells was observed. Both SIAIS125 and SIAIS126 inhibited the growth of H1975 cells (IC50 values of 19.4 nM and 30.8 nM, respectively) and PC9 cells (IC50 2.5 nM). Interestingly, they demonstrated that PROTAC-induced EGFR degradation was mediated by the ubiquitin/proteosome system and the ubiquitin/autophagy/lysosome system [66].

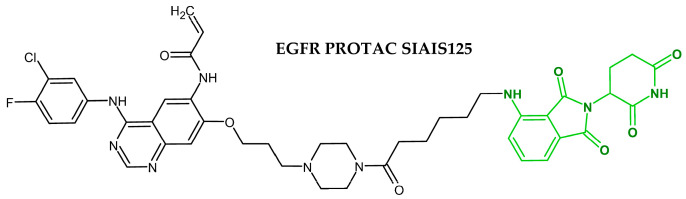



### 2.14. G1- to S-Phase Transition 1 and 2 (GSPT1/2)

GSPT1 and GSPT2 are small GTPases necessary for the transition from the G1- to the S-phase of the cell cycle. GSPT1/2 also act as a polypeptide chain, releasing factor 3 (eRF3), which promotes stop-codon recognition during translation and protein release from the ribosome. Acute leukemia cells are highly sensitive to GSPT1 degradation, making this protein a target for therapy for hematological cancers [67]. 

Nishiguchi et al. (2021) designed and synthesized a library of thalidomide analogues, and performed a screening of their activity against a panel of leukemia and medulloblastoma cell lines. They discovered a novel and potent GSPT1 molecular glue, sulfonamide 6 (SJ6986). After 24 h incubation, SJ6986 degraded GSPT1 in MV4-11 (AML) and MHH-CALL-4 cells, with DC50 values of 2.1 nM and 3.5 nM, respectively. The efficient depletion of GSPT1 by SJ6986 resulted in the induction of apoptosis and anti-proliferative activity against MV4-11 and MHH-CALL-4 cells, with EC50 values of 1.5 nM and 0.4 nM, respectively. Interestingly, this molecular glue did not affect the expression of IKZF1/3 or CK1α (prototypical IMiD neosubstrates), thus demonstrating selectivity toward GSPT1. The activity of SJ6986 was dependent on CRBN expression [68]. 

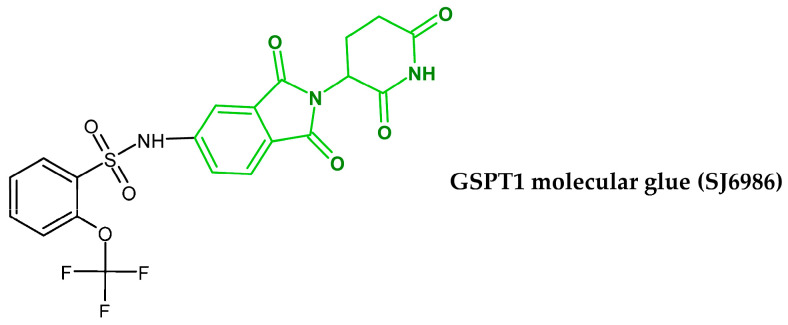



### 2.15. Transforming Growth Factor β1 (TGF-β1)

TGF-β1 is a pleiotropic cytokine involved in epithelial–mesenchymal transition (EMT), proliferation, cell differentiation, angiogenesis, extracellular-matrix production and cellular immunity. It plays an important role in tumor progression and the evasion of apoptosis by cancer cells, and leads to resistance to chemotherapy [69].

Feng et al. (2020) developed CRBN-recruiting PROTAC DT-6 composed of TGF-β1 inhibitor P144 and thalidomide. DT-6 efficiently degraded intracellular TGF-β1 via the proteasomal pathway in several cancer cells (THP1, BV2 leukemia, A549, MCF-7, U87 and HepG2) and slightly downregulated IKZF3 (the neosubstrate) in M2 macrophages. Furthermore, DT-6-induced degradation of TGF-β1 in M2 macrophages inhibited epithelial-to-mesenchymal transition (EMT) and the invasion of cancer cells [70].

### 2.16. Cytochrome P450 1B1 (CYP1B1)

CYP1B1 belongs to the heme-containing enzyme family, and is expressed in extrahepatic steroidogenic tissues and steroid-hormone target tissues. CYP1B1 activates many exogenous pro-carcinogens that are present in the environment, food or tobacco smoke (aromatic amines and polycyclic aromatic hydrocarbons). P450 1B1 is overexpressed in various types of human cancers such as breast, lung, esophageal, skin, testicular, colon, lymph-node and brain cancers. Moreover, it accelerates the inactivation of several anticancer drugs such as paclitaxel, docetaxel and doxorubicin, contributing to drug resistance [71].

Zhou et al. (2020) developed a series of PROTACs targeting CYP1B1 by coupling the thalidomide moiety to a derivative of α-naphthoflavone (ANF), a selective CYP1B1 inhibitor. PROTAC 6C with a six-carbon linker chain induced the degradation of CYP1B1 and restored sensitivity to docetaxel in the prostate-cancer cells overexpressing CYP1B1 (DU145/CY). Degrader 6C inhibited CYP1B1 significantly more weakly, compared to the ANF derivative (IC50 of 95.1 nM and 0.4 nM, respectively). The antiproliferative activity of degrader 6C and docetaxel against DU145/CY cells (IC50 7.01 nM) was comparable to that of docetaxel (IC50 4.95 nM) against parental DU145 cells [72].

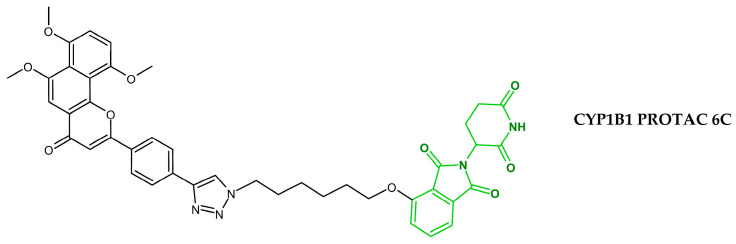



## 3. Factors Affecting the Efficacy of CRBN-Recruiting PROTACs

The mechanism of action of IMiD-PROTACs suggests that their clinical efficacy should be correlated with the expression of CRBN and other proteins forming the E3 ligase, and ubiquitin–proteasome system. Several in vitro studies confirmed that PROTAC activity depends on the CRBN and proteasome [40,46,73]. However, mechanisms regulating the effectiveness of IMiD-PROTACs in vivo are still unknown. Some insight is provided by clinical studies of multiple-myeloma patients refractory to IMiDs and by in vitro studies of cancer cell lines chronically treated with PROTACs.

### 3.1. Expression of CRBN, DDB1 and CUL4

IMiDs are used for the treatment of multiple myeloma (MM) or myelodysplastic syndrome (5q-MDS); however, prolonged treatment leads to reduced efficacy and drug resistance. The low expression of CRBN in MM patients is associated with poor response to the IMiDs. Schuster et al. observed no clinical response (i.e., stable or progressive disease) in patients with expression of CRBN below 0.8 (normalized RNA expression). However, 49% of patients with CRBN expression higher than 0.9 showed a partial or complete response to the IMiD treatment [74]. Patients with a high expression of CRBN showed improved progression-free survival (PFS) and overall survival (OS) in response to IMiDs [75,76]. Interesting results were published by Zhu et al., who observed that treatment with IMiDs led to the downregulation of CRBN in MM patients. An analysis of samples collected before the initiation of treatment and during relapse revealed that CRBN expression was reduced by 20–90%, resulting in resistance to IMiDs [77]. On the other hand, Dimopoulos et al. did not find a statistically significant correlation between clinical response to IMIDs and baseline levels of CRBN (probably due to a low number of patients analyzed). However, they observed a reduction in CRBN levels in patients treated with IMiDs [78]. 

Based on the above data, one can assume that the clinical efficacy of IMiD-PROTACs will be correlated with the level of CRBN. Patients with a high expression of CRBN are likely to show good clinical response to the treatment. Long-term therapy may reduce the expression of CRBN and decrease the effectiveness of IMiD-PROTACs, highlighting the need for development of degraders recruiting other E3 ligases (for example VHL or MDM2). Such an approach could resolve possible issues with loss of efficacy of IMiD-PROTACs, and would also provide alternative treatment for patients with low baseline expression of CRBN. 

The efficacy of IMiD-PROTACs may vary among different types of cancer, due to variations in CRBN expression. For example, staining of bone-marrow biopsies of MM patients demonstrated higher levels of CRBN in malignant plasma cells than in other hematopoietic cells. No staining was detected in the myeloma microenvironment (stromal cells) [78]. Higher expression of CRBN mRNA was observed in cancer cell lines originating from blood compared to solid tissues, suggesting that hematological cancers may respond better to IMiDs [74]. 

**Figure 2:**
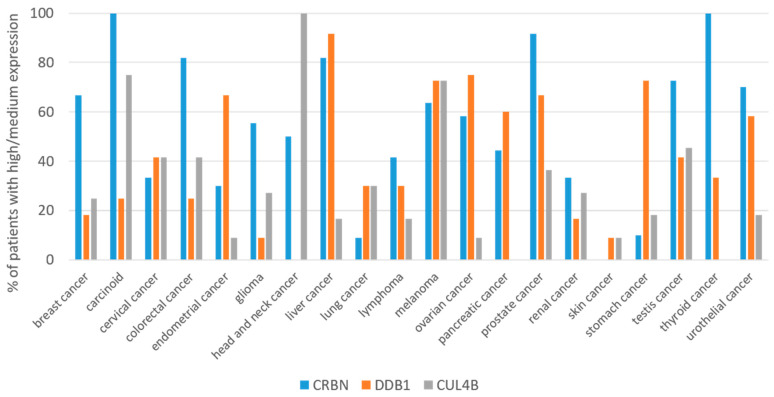
Expression of CRBN, DDB1 and Cullin 4B proteins in different cancer types. Fraction of patients with high/medium protein expression is shown. Data adapted from The Human Protein Atlas (https://www.proteinatlas.org/about/download; accessed on 20 October 2022).

According to The Human Protein Atlas [79], only 10% of patients with lung cancer express the CRBN protein at high/medium levels, whereas in prostate cancer this is about 90% of patients. Obviously, the expression of other components of CRBN E3 ubiquitin ligase, for example DDB1 or CUL4, could also affect the clinical efficacy of IMiD-PROTACs. Therefore, despite the relatively high expression of CRBN in patients with head and neck cancer and pancreatic cancer, they may respond poorly to IMiD-PROTACs, due to a lack of DDB1 and CUL4, respectively (Figure 2). This suggests that the high effectiveness of IMiD-PROTACs could be achieved in cancers with high expression of CRBN and other proteins of the E3 ligase. Assuming that the simultaneous, high expression of CRBN, DDB1 and CUL4 is crucial for the effectiveness of PROTACs, one can speculate that the use of PROTACs would be particularly beneficial in the treatment of melanoma, followed by prostate, cervical, testis and colorectal cancers. Currently, PROTAC CFT1946 (Table 1) has entered phase 1 and 2 of clinical trials, aiming to assess the safety and efficacy of CFT1946 in melanoma patients. This compound targets the BRAFV600X mutant kinase, which is present in approximately 60% of melanoma cases [80]. On the other hand, breast, endometrial, glioma, liver, lung, lymphoma, ovarian, renal, stomach and urothelial cancers would be less sensitive, as fewer than 20% of patients show high/medium expression of at least one of the E3 ligase components. The lowest efficacy can be expected in the case of head-and-neck, pancreatic, skin and thyroid tumors, due to the low level of expression of CRBN, DDB1 or CUL4.

### 3.2. Genetic Alterations of CRBN May Reduce the Efficacy of PROTACs

The downregulation of CRBN is not the only way that cancer cells may use to decrease the efficacy of IMiD-based degraders. Other mechanisms may include mutations in CRBN that impair interactions with IMiD-PROTACs or with DDB1, thus preventing the formation of the fully functional CRBN E3 ligase complex. Again, some insight is provided from the results of studies of patients resistant to immunomodulatory imide drugs. 

Several mutations in the CRBN gene were identified in 12% of patients refractory to IMiDs. These mutations include a nonsense mutation that result in the truncated protein without the C-terminal fragment containing the IMiDs binding domain. Other mutations, i.e., p.Ile393Metfs10X*, p.Phe381Cys, and p.Pro411His were located in the IMiDs binding domain, and presumably affect the drug binding. The introduction of these mutations into the MM cell line resulted in resistance to IMiDs [81]. Interestingly, none of these mutations concerned the amino-acid residues of CRBN directly involved in IMiD binding, i.e., His 378, Trp 380, Trp 386 and Trp 400 [82,83]. 

An increased frequency in CRBN mutations was observed in patients refractory to lenalidomide and pomalidomide, compared to untreated patients (mutations in 2.2%, 9% and 0.5% of patients, respectively). Patients refractory to lenalidomide and pomalidomide showed increased CRBN-gene copy loss, compared to untreated patients (detected in 7.9%, 24% and 1.5% of patients, respectively) [84].

Alternative CRBN splice variants with impaired IMiD binding may also promote resistance. Gandhi et al. identified splice variants lacking exon 10 encoding the portion of the IMID-binding domain (CRBN-E10) or without exons 5-7 (encoding the DDB1-binding domain) [85]. Interestingly, Gooding et al. confirmed the presence of the CRBN-E10 splice variant in the samples from MM patients, and found that its level was significantly increased in the pomalidomide-refractory patients [84].

These genetic alterations of CRBN (i.e., mutations, loss of the gene-copy and splice variants) were associated with resistance to IMiDs and worse clinical outcomes in MM patients. The above observations suggest that the long-term clinical use of IMiD-PROTACs can induce mutations in CRBN, impairing its stability and PROTAC binding.

## 4. Possible Resistance Mechanisms to PROTACs

The first evidence of the resistance to PROTACs was demonstrated in vitro in ovarian-cancer cells exposed to CRBN- and VHL-recruiting PROTACs targeting BET proteins. The resistance to CRBN-recruiting PROTACs was caused by the deletion of 12 Mbp fragment of chromosome 3 containing the CRBN gene leading to the downregulation of mRNA and protein. Interestingly, resistance to VHL-recruiting PROTACs was acquired by genetic alterations of Cullin 2 (CUL2), a scaffold protein in the VHL E3 ligase complex [86]. This raises an intriguing question as to whether cancer cells could develop resistance to IMiD-PROTACs by mutating other proteins composing the CRBN E3 ligase. 

The above observations were confirmed by Ottis et al., who obtained resistant clones of the acute myeloid leukemia cell line MV4-11 against CRBN- and VHL-recruiting PROTACs (dBet1, dBet6 and MZ1, respectively). DNA and transcriptome analysis revealed that resistance to IMiD-PROTACs resulted from the mutation in exon 4 of the CRBN gene, leading to a premature stop codon in mRNA and no detectable expression of protein. Other resistant clones showed no genetic mutations, but exhibited significant downregulation of the transcription of UBE2G1 (E2 ubiquitin-conjugating enzyme). Resistance of MV4-11 cells to VHL-recruiting PROTAC (MZ1) was acquired by the suppression of CUL2 transcription. The loss-of-function screen led to the identification of several genes involved in ubiquitination, whose inactivation might confer resistance to PROTACs. For CRBN-recruiting PROTACs, these are DDB1, UBE2G1 (E2 ubiquitin-conjugating enzyme), and, to a lesser extent, CUL4A. For VHL-recruiting PROTACs: VHL, UBE2NL, UBE2R2 (E2 ubiquitin-conjugating enzymes), ELO B/C (Elongin B and C), and COPS7A (a component of COP9 signalosome). The inactivation of genes for COPS8 (a component of COP9 signalosome) or RBX1 conferred resistance to both CRBN- and VHL-recruiting PROTACs [87].

Shirasaki et al. identified genes involved in resistance to CRBN- or VHL-recruiting PROTACs in a multiple myeloma cell line (MM1.S). The knockout of genes encoding CRBN and (to a lesser extent) DDB1 and UBE2G1, resulted in resistance to CRBN-recruiting PROTAC, while the knockout of Cullin 2 and VHL conferred resistance to VHL-PROTACs. The inactivation of genes encoding components of COP9 signalosome resulted in resistance to both CRBN- and VHL-recruiting PROTACs. Interestingly, they found that the overexpression of the gene-encoding multidrug resistance protein 1 (MDR1) resulted in the resistance of MM1.S cells to the VHL-PROTAC. This suggests that MDR1 (also known as P-glycoprotein) may be engaged in the removal of PROTACs from cancer cells [88].

The involvement of MDR1 in the resistance mechanism against PROTACs was confirmed by Kurimchak et al. They observed that high endogenous levels of the MDR1 protein in PROTACs-naïve cancer cells significantly reduced the efficacy of CRBN-recruiting dBet6 PROTAC, compared to the cells with low expression of MDR1. Genomic analysis of the ovarian-carcinoma A1847 cells resistant to CRBN- or VHL-recruiting PROTACs, revealed the amplification of the ABCB1 gene associated with increased expression of mRNA and protein. They also demonstrated that inhibitors of MDR1 pump (i.e., tariquidar) or the use of RTKs inhibitor lapatinib restored sensitivity of PROTAC-resistant cancer cells to protein degraders [89].

**Figure 3:**
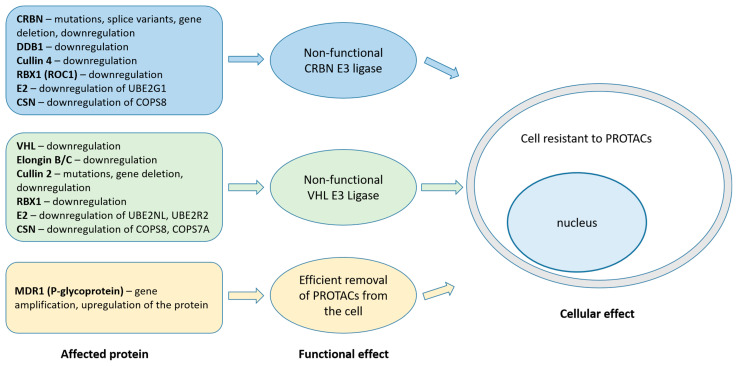
Possible mechanisms of resistance to CRBN- and VHL-recruiting PROTACs based on in vivo studies of patients refractory to IMiDs and in vitro studies of PROTAC-resistant cancer cells (for references, see the main text). Abbreviations: CSN—COP9 signalosome, E2—ubiquitin-conjugating enzyme, MDR1—multidrug resistance protein 1.

Interestingly, the above data suggest that mechanisms of resistance relate primarily to proteins that build the E3 ligase complex or to a drug efflux pump, rather than proteins targeted by PROTACs (Figure 3).

## 5. Conclusions

Preclinical studies have shown that PROTACs are highly effective in removing pathogenic proteins, and for this reason they entered the early stages of clinical trials. Most of the currently tested PROTACs recruit CRBN, and the results of ongoing clinical trials should provide information as to whether their efficacy is correlated with the expression of CRBN and/or other proteins composing the E3 ligase. In addition, these studies should indicate whether the expression of CRBN E3 ligase in tissue biopsies could serve as a predictor of a good clinical response to IMiD-PROTACs and thus warrant the development of validated methods for the measurement of CRBN E3 ligase expression.

It is very likely that long-term therapy with IMiD-PROTACs will trigger cellular mechanisms limiting the PROTACs efficacy or leading to resistance. These include the downregulation of CRBN (or CRBN E3 ligase), genetic alterations (mutations, gene deletion, alternative splice variants) or the upregulation of drug efflux pump(s). These challenges could be tackled by the development of PROTACs recruiting other E3 ligases (for example, VHL or MDM2). Such an approach could resolve issues of loss of efficacy or resistance to IMiD-PROTACs and also provide alternative treatment for patients with low baseline expression of CRBN.

## Figures and Tables

**Table 1:** PROTACs currently in clinical trials. Data from clinicaltrials.gov (https://clinicaltrials.gov/ct2/home; accessed on 20 February 2023). NA—not applicable.

PROTAC Name (Company)	E3 Ligase	Targeted Protein (Indication)	Trial Phase	Study Number
AC682 (Accutar Biotech Inc.)	CRBN	Estrogen receptor; (breast cancer)	Phase 1	NCT05489679; NCT05080842
ARV-471 (Arvinas)	CRBN	Estrogen receptor; (breast cancer)	Phase 1/2	NCT05501769; NCT04072952; NCT05549505; NCT05463952; NCT05573555; NCT05548127; NCT05732428
ARV-766 (Arvinas)	unknown	Androgen receptor; (prostate cancer)	Phase 1/2	NCT05067140
ARV-110 (Arvinas)	CRBN	Androgen receptor; (prostate cancer)	Phase 1/2	NCT05177042; NCT03888612
CC-94676 (Celgene/Bristol Myers Squibb)	CRBN	Androgen receptor; (prostate cancer)	Phase 1	NCT04428788
DT2216 (Dialectic Therapeutics)	VHL	BCL-X_L_; (Solid tumors, hematologic malignancy)	Phase 1	NCT04886622
FHD-609 (Foghorn Therapeutics)	CRBN	BRD9; (synovial sarcoma)	Phase 1	NCT04965753
CFT8634 (C4 Therapeutics)	CRBN	BRD9; (synovial sarcoma, soft tissue sarcoma)	Phase 1/2	NCT05355753
KT-474 (Kymera)	CRBN	IRAK-4; (atopic dermatitis, hidradenitis suppurativa)	Phase 1	NCT04772885
KT-413 (Kymera)	CRBN	IRAK-4; (non-Hodgkin lymphoma, diffuse large B-cell lymphoma)	Phase 1	NCT05233033
KT-333 (Kymera)	unknown	STAT3; (lymphomas, large granular lymphocytic leukemia, solid tumors)	Phase 1	NCT05225584
NX-2127 (Nurix Therapeutics)	CRBN	BTK; (B-cell malignancies)	Phase 1	NCT04830137
NX-5948 (Nurix Therapeutics)	CRBN	BTK; (B-cell malignances, chronic lymphocytic leukemia, Waldenstrom macroglobulinemia, primary central nervous system lymphoma)	Phase 1	NCT05131022
CFT1946 (C4 Therapeutics)	CRBN	BRAF^V600X^; (solid tumors, melanoma, non-small-cell lung cancer, colorectal cancer, anaplastic thyroid cancer)	Phase 1/2	NCT05668585
CG001419 (Cullgen)	CRBN	TRK—neurotrophic-factor receptor tyrosine kinase; (advanced solid tumors)	NA	FDA-approved for use in clinical trials.

## Data Availability

No new data were created.
